# The Validity and Reliability of Autism Behavior Checklist in Iran

**Published:** 2015-06

**Authors:** Negin Yousefi, Hooshang Dadgar, Mohammad Reza Mohammadi, Nahid Jalilevand, Mohammad Reza Keyhani, Azar Mehri

**Affiliations:** 1 School of Rehabilitation, Tehran University of Medical Sciences, Tehran, Iran; 2 Psychiatry and Psychology Research Center, Roozbeh Hospital, Tehran University of Medical Sciences, Tehran, Iran; 3 School of Rehabilitation, Iran University of Medical Sciences, Tehran, Iran; 4 Department of Basic Rehabilitation Sciences, Iran University of Medical Sciences, Tehran, Iran

**Keywords:** *Autism*, *Autism Behavior Checklist*, *Persian*, *Reliability*, *Validity*

## Abstract

**Objectives:** The aim of this study was to evaluate the psychometric features of the Persian version of the Autism Behavior Checklist (ABC).

**Method:** The International Quality of Life Assessment (IQOLA) approach was used to translate the English ABC into Persian. A total sample of 184 parents of children including 114 children with autism disorder (mean age =7.21, SD =1.65) and 70 typically developing children (mean age = 6.82, SD =1.75) completed the ABC. Internal consistency, test-retest reliability, concurrent and discriminant validity, and cut-off score were assessed.

**Results**: The results of this study revealed that the Persian version of the ABC has an acceptable degree of internal consistency (.73). Test–retest comparisons using interclass correlation confirmed the instrument’s time stability (.83). The instrument’s concurrent validity with Gilliam Autism Rating Scale (GARS) was verified; the correlation between total scores was .94. In the discriminant validity, the autism group had significantly higher scores compared to the normal group. Receiver Operating Characteristic (ROC) analysis revealed that individuals with total scores below 25 are less likely to be in the autism group.

**Conclusion:** The Persian version of the ABC can be used as an initial screening tool in clinical contexts.

Autism Spectrum Disorder (ASD) is a neurodevelopmental disorder characterized by deficits in social communication and social interaction, as well as restricted, repetitive patterns of behavior, interests or activities(1). In DSM-IV, autism spectrum disorders include autism disorder, Pervasive Developmental Disorder Not otherwise Specified (PDD-NOS), Asperger syndrome, Childhood Disintegrative Disorder (CDD) and Rett’s disorder (2), but in DSM-V, all of these disorders have been categorized under the term “autism spectrum disorder” (1). 

Several studies reported that the prevalence of ASD increased dramatically in recent years (3-8). These prevalence rates of ASD have a range from 250 per 10000 (9) to one in 88 children (4). Developing the new assessment instruments may be one factor that contributes to the raising of ASD prevalence. In addition, increase in the awareness about autism spectrum disorder may also contribute to this phenomenon (8, 10).

Early diagnosis of autism is crucial because a series of studies have demonstrated that early identification and early intervention leads to more positive outcomes in communication, social interaction and cognitive development (11-13).

There are some ASD screening tools such as Autism Behavior Checklist (ABC) (14), the Baby and Infant Screen for Children with Autism Traits (BISCUIT) (15), the Checklist for Autism in Toddlers (CHAT) (16), the Gilliam Autism Rating Scale (GARS) (17), the Childhood Autism Rating Scales (CARS) (18), the Autism Diagnostic Observation Schedule (ADOS) (19) and the Autism Diagnostic Interview-Revised (ADI-R) (20). However, there is only one validated instrument available in Persian for screening and assessing children with autism. Ahmadi et al. (201 1) investigated the psychometric features of the Gilliam Autism Rating Scale (GARS) for identifying children with autism in Iran. They used the GARS to interview 100 mothers of children with autism (mean age = 8.28 years) and 100 mothers of typical children. They reported that the GARS is a reliable instrument for screening and identifying children with autism (21).

The ASIEP-2 is a screening instrument used to evaluate children with autism and to create educational plans for these children. It consists of five subscales: the Autism Behavior Checklist (ABC), the Sample of Vocal Behavior, Interaction Assessment, Educational Assessment, and Learning Rate Prognosis (22).

Autism behavior checklist (ABC) is a well-established instrument used to screen and diagnose autism (23), and it has been used successfully in differential diagnosis of autism (24). Krug et al. (1980) selected the behavior items from nine sources: the criteria outlined by Kanner, Lovaas et al., the British Working Party’s Checklist or Creak’s Nine Points, Rimland’s Form E-2, the BRIAAC, Rendle- Short and Clancy’s Checklist, and Lotter’s Checklist (22). Also, it has been used by health professionals in various countries (25, 26). The ABC has been considered useful in the screening of these children (27, 28). ABC is a popular instrument for identifying children with autism spectrum disorder because of its simple use, scoring, and low cost (29).

The psychometric properties of the ABC have been investigated by Krug et al. (1980). They noted that in a new sample of 62 individuals with autism, 86% received total ABC scores within 1 SD from the standardization sample mean and the remaining 14% had scores within 1.5 SDs (14). The split-half reliability was also reported to be 0.87(14). 

In addition, Volkmar et al. (1988) reported the split-half reliability of the total scale to be 0.74 and split-half reliabilities of the subscales as ranging from 0.30 to 0.70 (28).

Sturmey et al. (1992) reported that in their study, coefficient alpha for the ABC total score was .87(30). The validity of the scale was also examined in a study carried out by Miranda-Linné and Melin (1997). They compared the total score of the scale between speaking and nonspeaking autistic individuals. The mean scores obtained in the two groups were both lower than the 68-point cutoff proposed by Krug et al. (1980). In their study, they confirmed the proposals of other authors to decrease the 68-point cutoff score, considering it too high to correctly identify children with autism, and suggested a cutoff score of 54 (31). 

In total, there is a crucial need to have valid and reliable screening instruments for children with autism spectrum disorders in Iran. Therefore, the purpose of this study was to translate ABC into Persian and establish the reliability and validity of this instrument in Persian. In addition, we used the Gilliam Autism Rating Scale (GARS) for examining ABC concurrent validity.

## Material and Methods

*Participants*

Using convenience sampling method, a total number of 189 parents of children aged 4 to 10 years old including 114 children with autism spectrum disorder (mean age = 7.21, SD = 1.65) and 70 typically developing children (mean age = 6.82, SD=1.75) participated in this study. T-test revealed no significant differences in the chronological age of the groups. Children with autism disorder were diagnosed by child psychiatrists according to DSM-IV Text revision; these children were recruited from 5 autism-specific schools in Tehran and from speech therapy clinics at Tehran University of Medical Sciences. Normally developing children were recruited from Tehran’s kindergartens and schools. Children with physical disorders, blindness, and deafness or with language disorders were excluded.

The study was approved by the Medical Ethics Committee of Tehran University of Medical Sciences.

*Instruments*

Autism Behavior Checklist

The ABC contains 57 items in five areas: Sensory, Relating, Body and Object use, Language, and Social and Self-help skills. Each item is scored from 1 to 4 and the total score is obtained by adding the weight of the different areas. Krug et al. (1980) assigned the cutoff point of 68 as a score to correctly classify children who were suspected of having autism; a score of 68 and above was associated with a high probability of clinical diagnosis. In the standardization sample, 90% of the sample who received ABC scores higher than 68 also had a previous diagnosis of autism. In contrast, 95% of the sample who received ABC scores lower than 53 were not diagnosed as autistic by clinicians (14).

*Gilliam Autism Rating Scale-2*

Gilliam Autism Rating Scale-2 is a behavioral checklist designed to identify autism in 3-22 year old individuals. GARS-2 contains 42 items involving three subscales: Stereotyped Behavior, Communication, and Social Interaction. Items are rated on a four point Likert scale ranging from never observed (0) to frequently observed (3). GARS is designed to be answered by parents or teachers. This scale does not need special training (32). Based on Cronbach Alpha, the reliability of the test indicates alpha coefficient of 0.89 in Iran (21).

*Procedure*

At first, permission to translate and evaluate the psychometric features of the ABC was obtained from Pro-Ed, the publisher of the instrument. The original version of the profile was translated into Persian according to International Quality of Life Assessment (IQOLA) approach. First, the checklist was translated into Persian language by two independent Persian professionals familiar with special education. The forward translations were compared and discussed in a group meeting of the two translators and two of the authors. Differences were discussed until consensus was reached about the final Persian version. Then, in order to examine the equivalence of this translated version with the original version, back-translation to English was done by a Persian-English bilingual professional. Third, a committee of 10 professionals including 6 speech and language pathologist and 4 child psychiatrists were asked to confirm the validity of the translation and made revisions to the Persian version. The Persian version of ABC was then administrated to 10 mothers (5 mothers of children with autism and 5 mothers of typically developing children) to provide a qualitative testing of readability and comprehension. This qualitative testing revealed no problem with the Persian Version. Like the ABC, the Persian adaptation consists of 57 items, which are divided into 5 subscales: Sensory, Relating, Body and Object use, Language, and Social and Self-help skills.

The purpose and procedure of the study were explained to all mothers, and written informed consent was obtained. 

The ABC was administered to all of mothers in the form an interview. The mothers answered yes or no regarding the presence of a given behavior. To examining concurrent validity between ABC and the GARS, the GARS was administered to only 45 mothers of verbal children with autism (mean age = 7.06, SD = 1.25), because the GARS has a communication subtest. Test-retest reliability was collected from 50 mothers of children with autism spectrum disorder (mean age = 7.25, SD = 1.62) with an interval of two weeks. 

*Data Analysis*

All statistical analyses were conducted using SPSS version 18.0. To calculate internal consistency, Cronbach’s alpha coefficient was used and test-retest was analyzed by interclass correlation. The concurrent validity of the ABC was evaluated by calculating the correlation coefficient between the total scores obtained from the ABC and the GARS. Discriminant validity of the ABC was assessed by performing independent t-test between the autism group and normally developing children. Receiver operating characteristic (ROC) curve analysis was used to determine optimal cutoff values for differentiation between autism and normally developing children based on the total score of ABC.

## Results

The internal consistency reliability of the items on the ABC was investigated using the Cronbach’s alpha coefficient. Cronbach’s alpha for the total score of 57 items was .73 and the item-total correlation ranged from 0.35 to 0.75 (Table 1). Internal consistency of the ABC subscales is displayed in Table 1.

*Subscale Correlations*

The correlation between each subscale with each of other four subscales is demonstrated in Table2. 

**Table 1:** Internal consistency (Cronbach's alpha) of the persian version of the ABC

**Variable**	**Number of items**	**Cronbach’s alpha**
Sensory	9	.44
Relating	12	.45
Body and object use	12	.49
Language	13	.67
Social skills	11	.35
Total	57	.73

**Table 2:** Subscale correlations of the persian version of the ABC

**Variable**	**Sensory**	**Relating**	**Body and object use**	**Language**	**Social skills**	**Total**
Sensory	1	.63*	.52*	.48*	.57*	.74*
Relating		1	.67*	.61*	.73*	.84*
Body and object use			1	.59*	.72*	.86*
Language				1	.57*	.79*
Social skills					1	.86*
Total						1

* Correlation is significant at the 0.01 level (2-tailed)

**Table 3:** Test-retest reliabilty of the persian version of the ABC

**Variable**	**Interclass Correlation**	**95% confidence Interval**		**Sig**
		**Lower Bound**	**Upper Bound**	
Sensory	.75	-.002	.938	.025
Relating	.51	-962	.879	.003
Body and object use	.87	.490	.969	.003
Language	.72	-.117	.931	.035
Social skills	.52	.225	.970	.005
Total	.83	.327	.958	.007

**Table 4:** Discriminant validity of the Persian version of the ABC

**Variable**	**ASD**		**ND**		**sig**
	Mean	SD	Mean	SD	
Sensory	8.82	5.25	1.03	1.18	.001
Relating	17.46	7.26	1.73	3.14	
Body and object use	14.78	8.28	1.28	2.52	.001
Language	12.79	7.28	1.03	2.43	.001
Social skills	12.37	4.88	2.23	3.02	.001
Total	66.22	19.84	7.12	9.29	.001

*Test-Retest Reliability* *:*

To determine the test-retest reliability of the ABC, we used Intraclass Correlation Coefficient (ICC) with two weeks interval. The stability was.83 (n = 20). The correlation between subscales is displayed in Table3.

 Concurrent Validity

The ABC and the GARS total scores were correlated at .94, and subscale correlations between instruments ranged from .37 to .92.

*Discriminant Validity*

To examine the instrument’s discriminant validity, independent t-test was performed between the two groups. Total scores and subscale scores were compared between children with autism spectrum disorder and normally developing children. There was a significant difference in total scores and subscale scores between the two groups; autism group had a significantly higher scores than the normal group (p<.001). These results are displayed in Table 4.

*ROC Curve*

Receiver operating characteristics (ROC) were examined to find optimal cut-off scores to indicate a diagnosis of autism. The optimal cut-off score for screening autism was 25, associated with .97 sensitivity and .98 specificity.

## Discussion

The purpose of this study was to evaluate the psychometric features of the Persian version of Autism Behavior Checklist (14). Reliability and validity were examined, and the cut-off point for the autism spectrum disorders was calculated. In the current study, Cronbach’s alpha was 0.73 for the total scale, similar to the internal consistency estimate of .74 reported by Volkmar et al. (28), and lower than that suggested by other authors which was .87 (14). Cronbach’s alpha for subscales ranged from 0.35 to 0.67 which is similar to that reported by Volkmar et al. which ranged from .30 to .70 (28).

Test–retest reliability of the ABC was .83 in this study which is acceptable and suggests that the results of the ABC are stable over time and enables the professionals to confidently interpret the results from the ABC.

The correlation between the total scores of the ABC and the GARS was .94, which confirmed the concurrent validity of the ABC with the GARS.

This study showed the validity of the ABC in discriminating children with autism from children with normal development. These results are in accordance with the original ABC development sample and other studies (14, 25, 27, 33).

The total score for the ABC is the sum of the five subscale scores. Higher scores indicate more autistic behavior symptoms (14). Using 68 as a cutoff score which was recommended by Krug et al. (14), only 46% of children with autism were classified as autistic. ROC analysis determined that individuals with the total scores below 25 are less likely to have autism. These results are in accordance with some researches which also have questioned the accuracy of the recommended cutoff scores and suggested to lower the cutoff score; these suggestions range from 39 to 54 (28, 31, 33-35). The cut-off score proposed by this study is lower than other studies. Krug et al. were concerned about the applicability of the ABC to high-functioning children with autism (14), and according to Yirmia, this instrument is not appropriate for use on school-age autistic children (35). Therefore, recruiting children from autism specific schools may make the obtained total scores lower. Second, all children used rehabilitation services and this led to the reduction of symptoms..

We suggest future studies to evaluate psychometric features of other screening and assessment tools for autism spectrum disorders in Iran.

## Limitation

The results of this study should be interpreted with the following main limitation. Because we used a convenience sampling method, representation of the general population was limited (36).

## Conclusion

According to these findings and similar to other studies, the ABC is not appropriate for school age children, but it can be used as an initial screening tool at clinics. For more accurate assessment, there is a need to use other valid and reliable instruments.
